# Mental health and wellness during COVID-19: Impact on healthy population

**DOI:** 10.1192/j.eurpsy.2021.746

**Published:** 2021-08-13

**Authors:** L. Lipskaya-Velikovsky

**Affiliations:** Occupational Therapy, Tel Aviv University, TelAviv, Israel

**Keywords:** Psychological Distress, COVID-19 lockdown, participation in daily life, quality of life

## Abstract

**Introduction:**

Pandemic outbreak brings multiple challenges into everyday life, with high potential to affect all aspects of health. It was previously demonstrated that epidemic is harmful to mental health (MH) of a whole population producing long-lasting and significant burden for the person and the society. However, such an impact was less investigated in COVID-19 pandemic.

**Objectives:**

Investigate aspects of MH among healthy population during Spring 2020 lockdown due to COVID-19; detect factors affecting MH and their cumulative effect on health-related quality of life (QOL).

**Methods:**

571 healthy volunteers completed electronic survey distributed through social networks. The survey contained standard tools for evaluation of (1) levels of stress, anxiety and depression, (2) objective and subjective parameters of participation in daily-life activities, (3) daily routines, (5) loneliness, (6) social connectedness, (7) self-efficacy and (8) quality of life.

**Results:**

We found high levels of stress, anxiety and depression among healthy population and low QOL in physical, psychological and social relationship domains. Employment, keeping daily routines, social connectedness, self-efficacy, enjoyment, satisfaction and meaning in daily-life activities were associated with better MH. All the aforementioned factors contributed significantly to QOL.

**Conclusions:**

COVID-19 outbreak rise multiple health issues, among them affected MH of the healthy, not infected population. Public health strategies should be implemented to mitigate impact of the COVID-19 pandemic on MH given its personal and social burden and its contribution to QOL. Addressing participation in daily life activities can be a useful tool to cope with impact of COVID-19 on MH.

